# Daily Habits in Parkinson's Disease: Validation of the Daily Habit Scale

**DOI:** 10.1002/mdc3.13863

**Published:** 2023-09-08

**Authors:** Dejan Georgiev, Asma Torkmani, Ruifeng Song, Patricia Limousin, Marjan Jahanshahi

**Affiliations:** ^1^ Department Clinical and Motor Neurosciences, Institute of Neurology University College London London United Kingdom; ^2^ Department of Neurology University Medical Centre Ljubljana Ljubljana Slovenia; ^3^ Artificial Intelligence Lab, Faculty of Computer and Information Sciences University of Ljubljana Ljubljana Slovenia

**Keywords:** daily habit scale, Parkinson's disease, habit formation

## Abstract

**Objective:**

The objective of the study was to validate a new scale for assessing habitual behavior—the Daily Habit Scale in patients with Parkinson's disease.

**Background:**

Parkinson's disease patients are impaired in habit learning and skill acquisition. Despite repeated practice, they have difficulty developing habitual responses.

**Methods:**

One hundred seventy‐nine patients (Median (Mdn) = 69 [64–76], 65 females) participated in the study. Corrected item‐to‐total correlations were calculated to assess the item‐convergent and item discriminant validity. Confirmatory factor analysis and assessment of internal consistency were also carried out. Concurrent validity in respect to measures of anxiety and depression, apathy, impulsivity, personality, multidimensional health locus of control, and health‐related quality of life was also calculated. To determine the test–retest reliability of the scale, 30 patients (Mdn = 69 [66–73], 9 females) completed a second copy of the scale 6 months after the first.

**Results:**

Twenty‐nine items (76%) and 9 items (24%) of the 38‐item scale, respectively, showed a very good and good convergent validity. All the items discriminated between their own factor and the other factors. The comparative fit index of 0.932 indicated an acceptable model fit of the data, whereas the root mean square error of approximation of 0.06 moderate model fit. The scale had a good internal consistency (Cronbach α = 0.792), and a moderate test–retest reliability (0.57). Females had higher scores on two factors compared to men (Factor 3: household activities and Factor 8: sleep‐related activities).

**Conclusions:**

The Daily Habit Scale is a reliable and valid tool to measure daily habits in Parkinson's disease.

## Introduction

Parkinson's disease (PD) is a chronic, neurodegenerative disorder characterized by a progressive loss of dopamine‐producing cells in the substantia nigra pars compacta and a consequent dopamine deficiency in the nigrostriatal dopamine system.^1^ In addition, in PD, there is a substantial loss of dopaminergic cells and their innervation in the posterior putamen^2^ that together with the supplementary motor area (SMA) control habit formation.^3^, ^4^, ^5^ Therefore, a feature of PD should be a difficulty in acquiring and relying on habitual behaviors.^6^ Basal ganglia (BG) dysfunction has been noted to impair the “automatic execution of learned motor plans” in PD.^7^

In confirmation, there is empirical evidence that PD patients are impaired in skill and habit learning tasks, such as, the serial reaction time or weather prediction task, which are acquired through repetition and practice.^8^, ^9^, ^10^, ^11^, ^12^ Despite repetition and practice, patients with PD have difficulty developing a habitual response.^13^ PD patients are also impaired in tasks that require learning from positive and negative feedback.^14^ Moreover, in PD patients, automatic control is gradually replaced by effortful, goal‐directed processing.^15^, ^16^ An example of this is walking. Walking is usually a well‐practiced behavior that is performed automatically and without much attention, and therefore, becomes habitual. In contrast, in PD, walking is no longer a habitual and automatic behavior, and patients pay attention to each step; therefore, a normally habitual behavior is performed in a goal‐directed fashion in PD.

PD is a progressive, multisystemic disorder. Motor, as well as non‐motor symptoms including cognitive decline,^17^ occur at different stages of the disease and progress over the years.^18^ This can substantially impact habit formation in PD, as habit formation relies on learning processes.^19^ Apathy, which is very common in PD, can also affect habit formation.^20^ Indeed, learning and motivation are closely related, and both are linked to dopamine.^21^ Dopaminergic degeneration leads to an impairment of both motivation and learning from feedback in PD.^21^ In addition, depression, which is also common in PD and sometimes difficult to distinguish from apathy^20^ can lead to altered patterns of habit formation.^22^ All these can also lead to loss of independence and to a consequently significant impairment of quality of life in PD.

Our daily lives are full of naturalistic habits related to self‐care (eg, brushing teeth and bathing), eating, smoking, drinking, and using the internet and sending/receiving emails. Recently, a new 38‐item Daily Habit Scale (DHS) was developed and validated in young healthy controls (HC).^23^ Unlike other previously available scales, such as the Self‐Reported Habit Index (SRHI)^24^ and the Creature of Habits Scale (COHS),^25^ the DHS allows quantification of not only frequency, but also automaticity, both of which define the strength of a habit. The scale covers a wide range of daily habits, and therefore, has the potential to be used in different populations, including PD. Apart from the well‐known negative association between smoking and PD, such naturalistic habits have never been studied in PD. Therefore, the objective of this study was to validate the DHS in PD patients.

## Methods

### Participants

PD patients attending a movement disorders outpatient clinic (PL) at the National Hospital for Neurology and Neurosurgery, London. Inclusion criteria for the patients who participated in the study were those with a clinical diagnosis of PD according to United Kingdom (UK) Brain Bank Criteria,^26^ whereas the exclusion criteria were those who had other major neurological, psychiatric, or physical diseases.

The questionnaires were sent out to 220 PD patients, of whom 179 responded (81% response rate) and were included in the study. A second copy of the DHS was mailed to 50 respondents (~28% of the test sample) 6 months after the initial completion to assess of the test–retest reliability of the new scale. This subgroup of patients was chosen randomly from the test sample using random number generator (minimal value, 1; maximal value, 179; 50 hits).^27^ Completed questionnaires were received for the assessment of test–retest reliability from 30 PD patients (60% response rate). Written informed consent was obtained from all participants in the study.

### Procedure

The ethics committee of University College London approved the study. All participants were mailed a booklet of questionnaires including the 38‐item DHS (see Supporting Information for a detailed description of the scale in Data S1),^23^ and all participants signed written consent before entering the study. Information on demographics (age, gender, handedness), medical history (disease duration, disease stage as assessed by Hoehn and Yahr scale,^28^ levodopa equivalent daily dose [LEDD])^29^ was also collected. In addition, the following validated generic PD specific questionnaires and scales were included in the booklet to assess the concurrent, convergent, and divergent validity of the DHS: (1) The Questionnaire for Impulsive‐Compulsive Disorders in Parkinson's Disease (QUIP)^30^ (anytime during‐PD full version), a global screening instrument for Impulsive‐Compulsive Disorders (ICD). (2) The Parkinson's Disease Questionnaire (PDQ‐8),^31^ a self‐administered questionnaire that reflects the impact of the motor and non‐motor symptoms of PD on patients’ quality of life. (3) The Hospital Anxiety and Depression Scale (HADS),^32^ a self‐report measure, to assess anxiety (HADS‐A) and half to depression (HADS‐D). (4) The Starkstein Apathy Scale (SAS),^33^ a self‐report instrument that screens for apathy and measure the severity of apathetic symptoms in PD. (5) The Barratt Impulsiveness Scale‐11 (BIS‐11),^34^ the most widely used self‐report instrument for impulsive personality/behavioral traits.^35^ (6) The Multidimensional Health Locus of Control (MHLC),^36^ a multidimensional self‐report instrument designed to assess an individual's belief in the locus of control regarding health behaviors and includes three factors—internal health locus of control (IHLC), powerful others health locus of control (PHLC), and chance health locus of control (CHLC). (7) The Tridimensional Personality Questionnaire (TPQ),^37^ which covers three personality dimensions—novelty seeking (NS), harm avoidance (HA), and reward dependence (RD). (8) Schwab and England Activities of Daily Living (ADL),^38^ which assesses the capabilities of people with impaired mobility. A detailed description of all scales and how the scores were calculated can be found in the Supporting Information.

### DHS Validation, Reliability Testing, and Statistical Analysis

The percentage of cases reporting engagement in each item in PD, the frequency of minimal and maximal responses on strength of habit as well as their skewness and kurtosis of both the test and retest‐sample are presented in Table S1A,B.

Multi‐trait scaling analysis was conducted by calculating the corrected item‐total correlation coefficients between the items and their corresponding factor and Spearman's ρ correlation coefficients between the items and other factors and used to assess the item‐convergent and item‐discriminant validity of the scale. In assessing item‐convergent validity, values of the corrected item‐to‐total correlation between 0 and 0.19 were taken to indicate poor convergent validity toward its own factor, values between 0.2 and 0.39 were taken to indicate good convergent validity toward its own factor, and values 0.4 and above were taken to indicate very good convergent validity toward its own factor.^39^ Items that correlated significantly higher (>1.96 standard errors) with its own factor that was hypothesized to measure than with the other six factors were considered as scaling successes in assessing item‐discriminant validity.

A confirmatory factor analysis (CFA) was conducted to assess the construct validity, that is, to determine whether the factor structure of DHS published before^23^ in healthy participants could be confirmed in data collected with PD patients. We evaluated the CFA results based on the comparative fix index (CFI) >0.9 and root mean square error of approximation (RMSEA) of <0.05 indicating a very good fit and a value between 0.05 and 0.10 indicating a moderate fit.^40^

Internal consistency was assessed with Cronbach α,^41^ and test–retest reliability was assessed with the interclass correlation coefficient (ICC).^42^ Next, the DHS factor structure^23^ was used to assess the concurrent validity by measuring the gender‐adjusted Spearman's ρ correlation coefficient of the underlying subscales of the DHS with the scales and subscales of HADS, SAS, TPQ, MHLC, BIS‐11, QUIP, PDQ‐8, and Schwab and England ADL. An α level of 0.05 or less was considered statistically significant. The false discovery rate^43^ was used to correct for multiple comparisons. The Mann–Whitney *U* test was used to compare DHS results between male and female patients and between the test and retest samples. The χ^2^‐test was used to compare gender and handedness distribution between the test and retest samples. Except for the CFA, which was conducted by the use of the software Ωnyx,^44^ the rest of the statistical analyses were performed using IBM SPSS v.26 for Mac.

## Results

### Participants

The demographic information and scores on the various measures are presented in Table 1. The PD test and retest samples did not differ with respect to disease duration and LEDD, or any other scales used to assess the patients (all *P* > 0.107), except for the PDQ‐8 score, which was significantly higher in the test sample than in the retest sample (*P* = 0.028).

**TABLE 1:** Demographic data and scores on the questionnaires/scales (median [25th–75th percentiles])

	PD test sample	PD retest sample	*P*
Age	69 (64–76)	69 (66–73)	0.883
Gender (f/m)	65/114	9/21	0.534
Handedness (r/l)	157/16	28/2	0.742
Disease duration	10 (5–15.25)	12.5 (4.75–17.5)	0.379
Hoehn andYahr	2 (1–3)	2 (1–2.75)	0.817
Schwab and England ADL	80 (60–90)	80 (60–90)	0.740
LEDD	590 (350–900)	655 (207.5–909)	0.703
HADS‐D	6 (3–8)	5.5 (2–7)	0.152
HADS‐A	6 (4–9)	5 (3.75–7.25)	0.107
SAS	19 (16.25–22)	19 (17–21)	0.418
TPQ – total	152 (143–162)	150 (144–175)	0.574
Novelty seeking	51 (47–54)	52.5 (48.75–54.5)	0.165
Harm avoidance	55 (52–59)	55 (52.5–48.75)	0.758
Reward dependence	44 (41–46)	43.5 (41–46)	0.647
MHLC – total	55 (48–62)	56 (46–60)	0.499
IHLC	18 (15–21)	17 (16–19.75)	0.708
PHLC	19 (15–24)	15 (13–22)	0.181
CHLC	19 (14–23)	18 (12.75–22)	0.524
BIS‐11 – total	63 (53–69)	63 (52–71)	0.978
Attention	15 (13–18)	13 (11–17)	0.202
Motor	21 (18.25–24)	21 (18–24)	0.918
Non‐planning	24 (20–29)	25 (20.25–29.5)	0.919
QUIP	1 (0–4)	0.5 (0–3)	0.393
PDQ‐8	10 (6–14)	7.5 (3–13)	0.028

Abbreviations: PD, Parkinson's disease; f/m, number of females/number of males; r/l, number of right handed/number of left handed; ADL, Activities of Daily Leaving; LEDD, levodopa equivalence daily dose; HADS‐A, Hospital Anxiety and Depression Scale, anxiety; HADS‐D, Hospital Anxiety and Depression Scale, depression; SAS, Starkstein Apathy Scale; TPQ, Three‐dimensional Personality Questionnaire; MHLC, The Multidimensional Health Locus of Control, PHLC, Powerful Others Health Locus of Control, IHLC, Internal Health Locus of Control; CHLC, Chance Health Locus of Control; BIS‐11, Barratt Impulsiveness Scale‐11; QUIP, Questionnaire for Impulsive‐Compulsive Disorders in Parkinson's disease; PDQ‐8, Parkinson's Disease Questionnaire‐8.

### Multi‐Trait Scaling Analysis

The results of the analysis are presented in Table 2. Regarding the item‐convergent validity, as mentioned earlier, values of the corrected item‐to‐total correlation between 0 and 0.19 were taken to indicate poor convergent validity toward its own factor, values between 0.2 and 0.39 were taken to indicate good convergent validity toward its own factor, and values 0.4 and above were taken to indicate very good convergent validity toward its own factor.^39^ In Factor 1 (hygiene and self‐care activities), five of seven items had a corrected item‐to‐total correlation higher than 0.4 and two had a value between 0.2 and 0.39. In Factor 2 (leisure activities), two of the six items had a value above 0.4, and four factors had a value between 0.3 and 0.39. In Factor 3 (household activities), all four items had a value above 0.4. In Factor 4 (common daily activities), five of six items had a value above 0.4, and one had a value of 0.379. In Factor 5 (unhealthy habits), five of seven items had a value above 0.4 and two items had a value between 0.2 and 0.39. In Factor 6 (sport‐related activities), Factor 7 (technology and internet use) and Factor 8 (sleep‐related activities) all items had a value above 0.4. Therefore, 29/38 (76%) items had corrected item‐to‐total correlations >0.4 indicating a very good convergent validity, and 9/38 (24%) items had corrected item‐to‐total correlations between 0.2 and 0.39 indicating good convergent validity. None of the items had an item‐to‐total correlation between 0 and 0.19 (poor convergent validity). All the items correlated significantly higher (more than 1.96 standard errors) with its own factor that is hypothesized to measure than with the other six factors indicating a scaling success in assessing item‐discriminant validity for all the items of the DHS.

**TABLE 2:** Corrected item‐to‐total correlation coefficients between the items and their corresponding factor (gray fields under the factor designations F1–F8) and Spearman's ρ correlation coefficients between the items and other factors

	F1	F2	F3	F4	F5	F6	F7	F8	SE	Cut‐off (−1.96 × SE)	Scaling success
F1: 19. Brushing my hair	0.693	0.183	0.344	0.345	0.096	0.244	0.051	0.169	0.072	0.553	Success
F1: 20. Washing my hands	0.627	0.170	0.431	0.388	0.202	0.239	0.078	0.212	0.062	0.505	Success
F1: 23. Taking bath/shower	0.608	0.230	0.376	0.321	0.242	0.220	0.122	0.197	0.053	0.504	Success
F1: 18. Brushing my teeth	0.572	0.187	0.351	0.321	0.146	0.165	0.136	0.225	0.052	0.469	Success
F1: 3. Drinking water	0.445	0.127	0.197	0.181	−0.016	0.063	0.023	0.179	0.051	0.346	Success
F1: 22. Putting on makeup/perfume	0.315	0.126	0.292	0.245	0.007	0.155	0.161	0.233	0.035	0.245	Success
F1: 21. Shaving	0.233	0.073	0.033	−0.049	0.169	0.132	0.028	−0.111	0.040	0.154	Success
F2: 30. Surfing internet	0.155	0.641	0.259	0.074	0.338	0.234	0.696	0.158	0.081	0.482	Success
F2: 38. Playing games	−0.046	0.496	0.061	0.043	0.117	0.147	0.273	0.018	0.061	0.376	Success
F2: 35. Listening to music	0.274	0.386	0.205	0.319	0.338	0.242	0.097	0.114	0.037	0.314	Success
F2: 27. Watching TV	0.290	0.373	0.068	0.251	0.130	0.110	0.086	0.085	0.041	0.294	Success
F2: 11. Having rest	0.061	0.323	−0.008	0.069	0.005	0.039	−0.051	0.192	0.043	0.238	Success
F2: 5. Chewing gum	0.024	0.314	0.168	−0.018	0.092	0.013	0.230	0.056	0.041	0.233	Success
F3: 16. Carrying out household chores	0.418	0.207	0.761	0.302	0.306	0.277	0.234	0.057	0.073	0.619	Success
F3: 17. Cleaning/tidying	0.386	0.234	0.753	0.268	0.280	0.259	0.278	0.059	0.070	0.615	Success
F3: 15. Gesturing	0.194	0.143	0.650	0.265	0.077	0.158	0.092	0.173	0.065	0.523	Success
F3: 24. Emptying bowels	0.457	0.244	0.479	0.331	0.194	0.228	0.214	0.066	0.049	0.383	Success
F4: 10. Taking pain killers	0.277	0.108	0.157	0.613	0.050	0.028	0.016	0.160	0.069	0.477	Success
F4: 34. Reading newspaper	0.317	0.224	0.313	0.608	0.153	0.242	0.070	0.080	0.061	0.489	Success
F4: 9. Taking my medication prescribed	0.358	0.201	0.161	0.478	0.124	0.084	−0.019	0.209	0.055	0.370	Success
F4: 25. Shopping	0.223	0.268	0.344	0.457	0.345	0.209	0.259	0.142	0.035	0.389	Success
F4: 4. Drinking coffee/tea	0.214	0.043	0.198	0.379	0.092	0.282	0.039	0.067	0.044	0.293	Success
F5: 8. Eating savory snacks/crisps	0.047	0.306	0.098	0.096	0.610	0.037	0.161	0.068	0.069	0.475	Success
F5: 14. Having sex	0.104	0.152	0.110	0.047	0.545	0.138	0.140	0.004	0.059	0.430	Success
F5: 6. Eating fast food	−0.023	0.155	0.078	0.106	0.487	0.072	0.108	0.107	0.053	0.383	Success
F5: 7. Eating chocolate/sweets	0.087	0.266	0.084	0.099	0.472	0.001	0.061	0.103	0.054	0.367	Success
F5: 2. Having alcoholic drink	0.157	0.134	0.211	0.162	0.454	0.151	0.168	−0.068	0.050	0.356	Success
F5: 26. Spending money	0.255	0.314	0.282	0.276	0.358	0.385	0.300	0.111	0.029	0.301	Success
F5: 1. Smoking	0.082	0.026	0.049	0.065	0.202	−0.067	0.151	−0.030	0.031	0.141	Success
F6: 36. Exercising	0.328	0.072	0.244	0.279	0.058	0.651	0.022	−0.002	0.078	0.499	Success
F6: 29. Calling particular people	0.184	0.224	0.166	0.173	0.147	0.618	0.309	0.203	0.055	0.510	Success
F6: 32. Socializing	0.269	0.340	0.409	0.350	0.272	0.600	0.239	0.169	0.046	0.509	Success
F6: 37. Participating in sports	0.022	0.083	−0.001	−0.089	0.046	0.490	0.172	0.010	0.063	0.367	Success
F7: 28. Using my mobile phone	0.051	0.421	0.240	0.084	0.265	0.292	0.820	0.123	0.087	0.649	Success
F7: 31. Checking email	0.211	0.551	0.275	0.134	0.307	0.273	0.797	0.145	0.080	0.640	Success
F7: 33. Social networking	0.055	0.294	0.097	0.133	0.169	0.116	0.594	0.179	0.061	0.474	Success
F8: 12. Sleeping late	0.115	0.155	0.030	0.107	0.062	0.160	0.119	0.654	0.070	0.517	Success
F8: 13. Waking up early	0.204	0.181	0.124	0.127	0.117	0.019	0.099	0.552	0.057	0.440	Success

*Note*: F1, hygiene and self‐care activities; F2, leisure activities; F3, household activities; F4, common daily activities; F5, unhealthy habits; F6, sport related activities; F7, technology and internet use; F8, sleep‐related activities; SE, standard error of the correlations.

*Note*: The cutoff value was calculated as the differences between the value of the corrected item‐to‐total correlation for the respective item and the product of 1.96 of the SE for the same item. The scaling success was defined as a success if the value of the corrected item‐to‐total correlation was higher than the cutoff value. The items belonging to different factors are presented in the first column.

### Confirmatory Factor Analysis

The CFI index was 0.934 indicating acceptable model fit, and the RMSEA was 0.06, indicating a moderate model fit of the data to DHS.

### Internal Consistency and Test–Retest Reliability

The overall Cronbach's coefficient α was 0.792. The ICC = 0.57, *P* < 0.001, testing for the test–retest reliability, was moderately high. In addition, the correlations between the factors in the test and retest samples were all significant (Table S2A,B, respectively).

### Concurrent Validity

A correlation analysis was performed between the factor scores of the DHS and measures of anxiety, depression, apathy, trait impulsivity, and other personality dimensions, and health‐related quality of life for the PD patients. For PD patients, depression correlated negatively with Factor 3 (household activities), ρ = −0.22, *P* = 0.003, Factor 6 (sport‐related social activities), ρ = −0.26, *P* = 0.001, and Factor 7 (technology and internet use), ρ = −0.16, *P* = 0.034 and positively with factor Factor 5 (unhealthy habits) ρ = 0.21, *P* = 0.007. Anxiety did not correlate significantly with any of the factors (all *P* > 0.206). Apathy correlated positively with Factor 7 (technology and internet use), ρ = 0.19, *P* = 0.011. Health‐related quality of life correlated negatively with DHS Factor 3 (household activities) ρ = −0.21, *P* = 0.008.

Factor 1 (hygiene and self‐care activities) correlated negatively with the total trait impulsivity score as measured by BIS‐11, ρ = −0.23, *P* = 0.015. Although the BIS‐11 attentional subscale and BIS‐11 motor subscale did not correlate with any of the factors, the BIS‐11 non‐planning scale correlated with Factor 1 (hygiene and self‐care activities) ρ = −0.18, *P* = 0.036 and Factor 7 (technology and internet use) ρ = −0.19, *P* = 0.028. The QUIP did not correlate significantly with any of the DHS factors (all *P* > 0.058). The total MHCL score correlated negatively with Factor 7 (technology and internet use), ρ = −0.23, *P* = 0.005. Although the IHLC subscale correlated positively with Factor 1 (hygiene and self‐care activities), ρ = 0.19, *P* = 0.016, Factor 3 (household activities), ρ = 0.23, *P* = 0.003 and Factor 6 (sport‐related activities), ρ = 0.24, *P* = 0.002, the PHLC subscale negatively correlated with Factor 5 (Unhealthy habits), ρ = −0.21, *P* = 0.013 and Factor 7 (technology and internet use), ρ = −0.36, *P* < 0.001. The CHLC subscale did not correlate significantly with any of the factors (all *P* > 0.107). There was no significant correlation between the measures of personality as assessed by the total TPQ and the TPQ subscales with any of the DHS factors (all *P* > 0.069). Schwab and England ADL scale positively correlated with Factor 2 (leisure activities) ρ = 0.15, *P* = 0.045, Factor 3 (household activities) ρ = 0.35, *P* < 0.001, Factor 5 (unhealthy habits) ρ = 0.29, *P* < 0.001, Factor 6 (sport‐related activities) ρ = 0.25, *P* < 0.001 and Factor 7 (technology and internet use) ρ = 0.26, *P* = 0.001.

### Differences between Males and Females

**FIG. 1:**
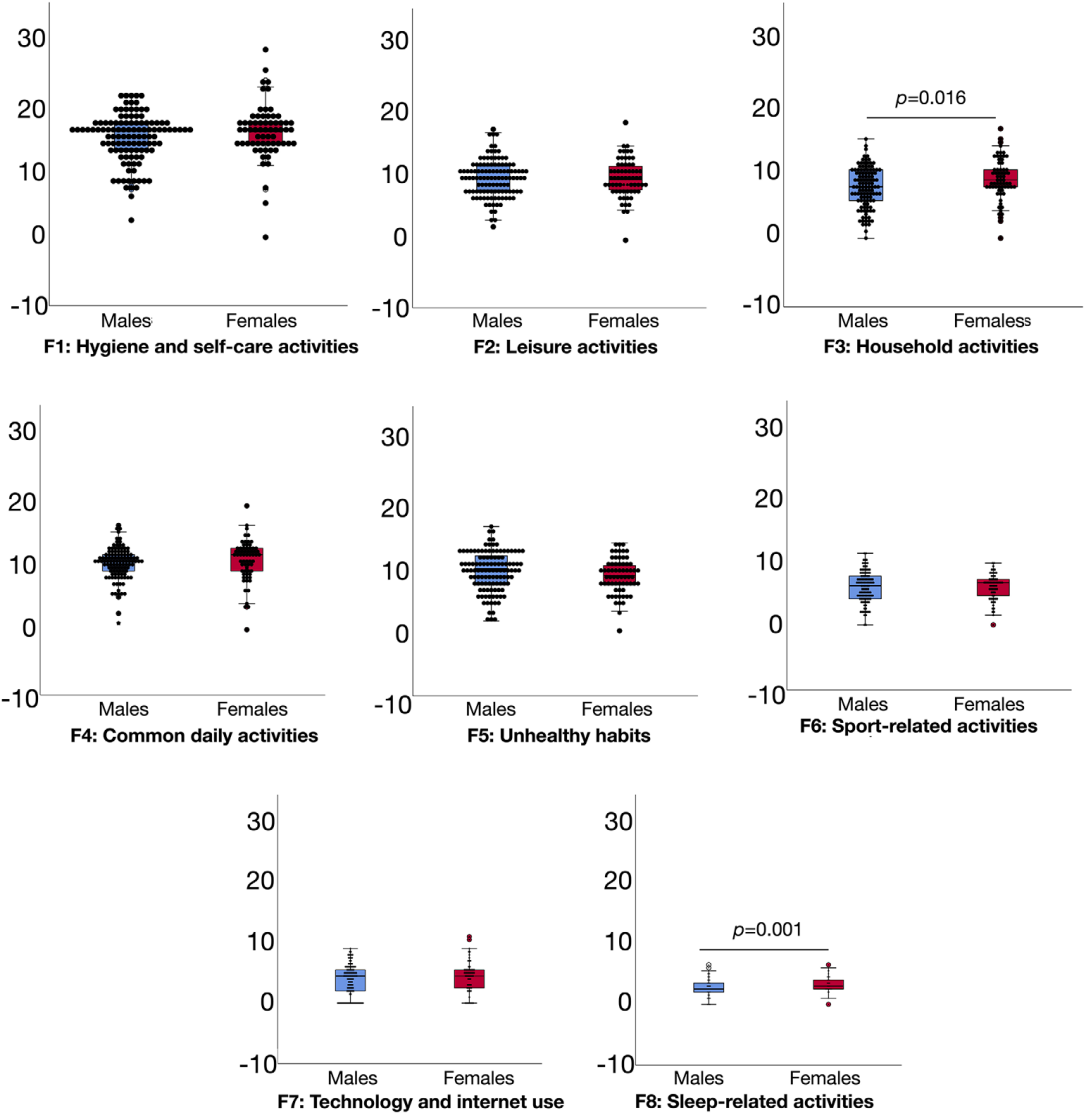
Boxplots (95% confidence interval, 25%–75% percentiles, median) of the factors of the Daily Habit Scale Factors (y‐axis) in males (n = 114) and female (n = 65) patients with PD (x‐axis).

The differences between male and female patients are presented in Figure 1. Females (Median (Mdn) = 8.5 [7.5–10.5]) scored significantly higher than males (Mdn = 7.5 [5.5–10.0]) on Factor 3 (household activities), *Z* = −2.41, *P* = 0.016, and Factor 8 (sleep‐related activities), Mdn = 3 (2.5–4) and Mdn = 2.5 (2.0–3.5) for females and males, respectively, *Z* = −3.17, *P* = 0.001. There were no significant differences in the other factors (all *P* > 0.112).

## Discussion

The aim of this study was to validate the DHS in PD. The results of the study showed that the DHS is a valid and reliable scale for measuring daily life habits in PD patients.

### Item Convergent and Item Discriminant Validity, Confirmatory Factor Analysis, Internal Consistency, and Test–Retest Reliability of DHS in PD

PD has a major impact on patients’ daily activities. The motor and non‐motor impairments lead to disability in performing and fulfilling daily tasks.^45^, ^46^ To date, there are no specific scales to measure the extent of impairment of daily habits in patients with PD. Although the SRHI^24^ has good test–retest reliability, it is complicated to complete because it includes a 12‐item questionnaire and a 7‐point Likert scale, as well as a behavioral sheet. It is also repetitive, for example, Item 4 (“That makes me feel weird if I do not do it”) is almost identical to Item 6 (“That would require effort not to do it”). The COHS^25^ consists of 27 questions divided into two subscales measuring routine behavior and automatic responses on a 5‐point Likert scale. However, this questionnaire relates more to self‐perception of habitual actions in the general population and does not allow for adequate quantification of habits. We recently developed and validated the DHS,^23^ which consists of 38 items. The results showed that the DHS is a valid and reliable scale for measuring daily habits in young, healthy people. In the current study, we validated the scale in PD. The primary step in assessing whether DHS can be used to assess habits in PD was the multi‐trait scaling analysis. The analysis showed that 29/38 items (76%) had a very good convergent reliability, and 9/38 items (24%) a good convergent reliability. All the items successfully fulfilled the criterion of discrimination between item's own factors and other factors.

CFA is considered a good way of assessing construct validity of scales. It necessitates a strong prior theory underlying the model before analyzing the data. The eight factor structure of DHS has already been established in a large sample of healthy young adults.^23^ Therefore, we used CFA to assess the construct validity of DHS in the present study. The results of the CFA showed acceptable/moderate model fit of the eight‐factor DHS structure to the PD data, confirming the construct validity of DHS in PD patients. Internal consistency relates to the extent to which all the items of a scale measure the same concept or construct.^41^ In this study, a Cronbach α = 0.792, indicating a good level of internal consistency of the DHS applied to PD patients. The test–retest reliability reflects the variation in measurements taken by an instrument on the same participant under similar conditions. In our study, the test–retest reliability based on ICC of 0.57 was moderately high.^42^

### Concurrent Validity of the DHS in PD

There were significant correlations between some of the mood, behavior, and personality measures and the DHS. Because depression is associated with impaired reward processing and social functioning, this could lead to impaired habit formation.^22^ In our study, this was reflected in the negative association of depression with household activities, sport‐related activities, and technology and internet use. The relationship between physical activity and depression is quite complex. On the one hand, depression may lead to decreased physical activity, and consequently, for example, reduced level of household and sport‐related activities. On the other hand, physical activity,^47^ especially sport‐related activity, leads to an improvement in depressive symptoms. The negative correlation between technology and internet use and depression is surprising, as data suggest that people who spend a lot of time on the internet are more likely to have depressive symptoms.^48^ However, internet and technology use are also age‐dependent, as older people tend to use the internet and other technologies less than younger people.^49^ There was a positive correlation between depression and unhealthy habits. Namely, studies have shown that depressive subjects frequently smoke, have a higher prevalence of excessive alcohol drinking and are more physically inactive.^50^ Apathy is very common in PD and can severely affect quality of life.^51^ Apathy depends on the normal functioning of networks that include the BG, prefrontal cortex, and the limbic system. Structural and functional changes in these networks could lead to abnormalities in goal‐directed behaviors, and therefore, to apathy.^51^ Surprisingly, there was a positive correlation between apathy and technology and internet use. A possible explanation could be that patients with apathy still perform highly structured and habitual tasks, such as technology and internet use, but fail to engage in other activities that require more effort. The factor technology and internet use from DHS consists of three items: using mobile phone, checking email, and social networking. Social media have already been linked to increase of apathy by providing a passive alternative to active participation.^52^ Another explanation would be that the use of mobile phones and checking emails reflect an attempt by apathetic patients to overcome their apathy to an extent. Regarding health‐related quality of life, PDQ‐8 negatively correlated with household activities, including cleaning/tiding. These important activities of daily living are very often perceived as a burden by PD patients and seem to negatively affect their quality of life.^53^, ^54^

As for the correlations between impulsivity and DHS in PD, there was no significant correlation between attentional and motor impulsivity to any of the factors of the DHS. The negative correlation between non‐planning impulsivity and hygiene and self‐care activities, and technology and internet use that initially appears surprising, in fact seems plausible because it could lead to less attention to hygiene and other self‐care activities, and less engagement in activities, social networking, and checking emails. In our study, the QUIP did not correlate with any of the factors of the DHS. The reason for this is that the QUIP assesses specific impulsive and compulsive behaviors usually related to dopaminergic medication use, such as hypersexuality, gambling, or compulsive use of dopaminergic medication,^30^, ^55^ whereas the DHS assesses habitual behaviors related to everyday activities. Therefore, pathological habits as measured by the QUIP and daily habits assessed by the DHS appear to be distinct. Similarly, no significant correlations were found between the DHS and its factors with personality assessed on the TPQ, suggesting that the DHS is mainly related to mood and is not strongly influenced by the dimensions of personality tapped by the TPQ.

Regarding health locus of control,^36^ the factors hygiene and self‐care activities, household activities, and sport‐related activities showed a positive correlation with IHLC, as higher IHLC scores suggest that subjects are more likely to believe that health is the result of their own behavior.^36^, ^56^ Moreover, leisure activities, household activities, unhealthy habits, and technology and internet use correlated negatively with PHLC, as higher scores on the PHLC indicate that people believe that their behavior depends on others (eg, physicians).^36^, ^56^ In contrast to IHLC, which was higher in HCs, PHLC was higher in PD. No significant correlations were found between CHLC (implying that people believe their health depends on chance) and DHS factors. We also found important correlations between the Schwab and England ADL and leisure activities, household activities, unhealthy habits, sport‐related activities, and technology and internet use. The Schwab and England ADL is a robust scale that assesses the capabilities of people with impaired mobility including PD.^38^ The higher the score, the more independent and able the person is to engage in different activities, including leisure activities, doing things at home, playing sports, using technology, but also adopting unhealthy habits.

### Gender Differences in DHS in PD

We found significant differences between male and female patients on two factors of the DHS. Namely, females scored higher on household activities and sleep‐related activities. Regarding household activities, it seems that women still predominate when it comes to performing activities related to home maintenance, such as cooking and cleaning. The sleep‐related activities factor of the DHS consists of two elements: sleeping late and waking up early. Studies have shown that women are at increased risk of insomnia,^57^ but they have also been shown to sleep more and better compared to men. They also tend to be more able to cope with an increase in cytokines when sleep is lost, which may explain the association of female gender with the second item (waking up early) of the sleep‐related activities factor of the DHS.

### Limitations of the Study

There are several limitations to the study. The CFA based on CFI and RMSEA showed only an acceptable/moderate fit for the model.^58^ Although some studies suggest that a level of CFI 0.95 is required for a model to be considered a good fit,^58^ most studies still use CFI level of more than 0.9 as a good fit for the model.^40^ Similarly, an RMSEA value of <0.05 is considered to indicate a very good fit for the model, whereas values between 0.05 and 0.1 indicate a moderate fit for the model.

The items of the DHS do not include important activities such as administrative work, paying bills, making appointments, and activities related to gainful or ungainful employment. Although these activities are important, some such as employment‐related activities or making appointment or paying bills are more goal‐oriented than habitual. The DHS with its 38 items is comprehensive and captures most daily habitual activities. In addition, some of the items, such as spending money, may include paying bills.

The retest was administered 6 months after the first test. Although the most commonly recommended time interval between test and retest is 2 weeks, the optimal time interval varies depending on the construct being measured and the stability of the construct over time.^59^ Indeed, habits are well‐formed and stable activities that change little over time, even though the target group is patients with PD, a progressive disease that can alter the ability to perform habits.

In summary, we validated the DHS consisting of 38 items distributed across eight factors, for testing the basic, daily habits in PD. The usefulness of this questionnaire remains to be further assessed in future clinical studies testing habitual performance in PD. By providing an opportunity to identify abnormalities in habitual behavior in these eight domains, this scale can potentially help to better understand the mechanisms of habit formation and reliance on daily habits in PD.

## Author Roles

(A) Design, (B) Execution, (C) Analysis, (D) Writing, (E) Editing of final version of the manuscript.

D.G.: A, B, C, D, E

A.T.: A, E

R.S.: A, B, C, E

P.L.: B, E

M.J.: A, B, C, D, E

## Disclosures

**Ethical Compliance Statement:** The ethics committee of University College London approved the study. All subjects signed written consent before entering the study. We confirm that we have read the Journal's position on issues involved in ethical publication and affirm that this work is consistent with those guidelines.

**Funding Sources and Conflicts of Interest**: No specific funding was received for this work. The authors declare that there are no conflicts of interest relevant to this work.

**Financial Disclosures for the Previous 12 months**: The authors declare that there are no additional disclosures to report.

## Supporting information


**Data S1.** Supplementary Methods. Detailed description of the scales.

**Table S1.**(**A**) Frequency (%) of all responses, minimal (min.) and maximal (max.) values on strength (S = (F + A)/2), frequency of minimal and maximal responses on strength, and skewness and kurtosis of the responses on strength for the PD test sample (N = 179). (**B**). Frequency (%) of all responses, minimal (min.) and maximal (max.) values on strength (S = (F + A)/2), frequency of minimal and maximal responses on strength, and skewness and kurtosis of the responses on strength for the PD retest sample (N = 30).
**Table S2.** (**A**) Factor by factor Spearman ρ correlation coefficients and the respective *p*‐values based on the PD test sample (N = 179). (**B**) Factor by factor Spearman ρ correlation coefficients and the respective *p*‐values based on the PD retest sample (N = 30).
