# Supplementary material for: Daily Habits in Parkinson's Disease: Validation of the Daily Habit Scale
Source: Mov Disord Clin Pract. 2023 Sep 8;10(10):1485–95. doi: 10.1002/mdc3.13863 (PMC10585975; doi:10.1002/mdc3.13863)
Supplement: Supplementary file 1 — Data S1. Supplementary Methods. Detailed description of the scales. [file MDC3-10-1485-s001.docx]

**Supplementary methods**

Detailed description of the scales

1. The Daily Habit Scale (DHS) ^1^ assesses naturalistic habits by evaluating engagement in daily habits. It contains 38 items. Responses for all items are closed-ended and each begins with two options: *I do not do at all* (go to next question) or *I do* (answer the questions bellow) on a four-point scale *I do several times a day/ daily/ weekly/ monthly* (please circle the choice), which allows quantifying the frequency (F) but also the automaticity (A) of a habit: *I do automatically/without thinking, I start doing before I realize I am doing it, I would find hard not to, I would continue doing regardless of any consequence.* For each item, frequency was scored from 0 to 5 (0 = I do not do at all, 1 = I do, 2 = I do monthly, 3 = I do weekly, 4 = I do daily, 5 = I do several times a day). Each of the four applicable automaticity options is given a score of 1 which are added together. From F and A, the strength of the habit (S) was calculated as a mean of these two values [S=(F+A)/2]. A total strength score for the DHS ranging from 0 to 171 is calculated by adding the strength for each item. A higher score indicates a higher level of daily habitual behaviour.

2. The Hoehn and Yahr (H&Y) scale ^2^ is a commonly used system for describing the symptoms of Parkinson's disease and how they progress. It consists of five stages: Stage 1: Unilateral involvement only usually with minimal or no functional disability, Stage 2: Bilateral or midline involvement without impairment of balance, Stage 3: Bilateral disease: mild to moderate disability with impaired postural reflexes; physically independent, Stage 4: Severely disabling disease; still able to walk or stand unassisted and Stage 5: Confinement to bed or wheelchair unless aided.

3. The Questionnaire for Impulsive-Compulsive Disorders in Parkinson’s Disease (QUIP) ^3^ (anytime during-PD full version) is a global screening instrument for Impulsive-Compulsive Disorders (ICD), which has 3 sections: section 1 assesses four impulse control disorders (gambling, hypersexuality, buying and eating behaviours); section 2 other compulsive behaviours (punding, hobbyism and walkabout); and section 3 is compulsive medication use ^3^. For the ICD section, the number of endorsed items for a positive screen is: (1) compulsive gambling: affirmative answers are any 2 or more out of the 5 gambling items; (2) compulsive sexual behaviour: any 1 or more out of the 5 sexual behaviour items; (3) compulsive buying: any 1 or more out of the 5 buying items; (4) compulsive eating: any 2 or more out of 5 eating items. For section 2 other behaviours, the criteria were: (1) Hobbyism: item #1A; (2) Punding: item #1B; (3) item #1C. For section 3, medication use, the criteria were item #1 and item #4. For the purposes of this study, each affirmative answer was marked with 1. A total score was then calculated by summing the responses for individual items. This questionnaire was administered to PD patients only.

4. The Parkinson’s Disease Questionnaire (PDQ-8) ^4^ is a self-administered questionnaire that reflects the impact of the motor and non-motor symptoms of PD on patients’ quality of life. It consists of 8 questions assigned to eight dimensions of health-related quality of life and daily activities as adversely affected by PD ^4^. The eight dimensions include Mobility, Activities of daily living, Emotional wellbeing, Stigma, Social support, Cognition, Communication, Bodily discomfort. It is scored on a scale of 0 to 100, with lower scores indicating better health and high scores indicating more severe symptoms. This questionnaire was administered to PD patients only.

5. The Hospital Anxiety and. Depression Scale (HADS) ^5^ is a self-report measure, consisting of 14 items, half of which relate to anxiety (HADS-A) and half to depression (HADS-D). Each item is scored from 0 to 3 in terms of severity/frequency ^5^. The total scores for each of anxiety and depression range from 0 to 21, with higher scores indicating more severe mood disturbance. Scores of 0-7 are in the normal range, 8-11 are borderline, and 11-21 are indicative of ‘caseness’.

6. The Starkstein Apathy Scale (SAS) ^6^ is a 14-item self-report instrument that screens for apathy and measure the severity of apathetic symptoms in PD. The scale consists of items related to diminished motivation (items 7 and 12), behavioural (items 4, 5, 8 and 9), cognitive (items 1, 2, 6 and 11), emotional (items 10 and 13) and insight components of apathy (items 3 and 4). The total score range is 0-42 and the higher the score, the more severe the apathy.

7. The Barratt Impulsiveness Scale -11 (BIS-11) ^7^ includes 30 items, and is the most widely used self-report instrument for impulsive personality/behavioural traits ^8^. There are six first-order factors, including attention (5, 9*, 11, 20 and 28), motor (2, 3, 4, 17, 19, 22 and 25), self-control (1*, 7*, 8*, 12*, 13* and 14), cognitive complexity (10*, 15*, 18, 27 and 29*), perseverance (16, 21, 23 and 30*) and cognitive instability (6, 24 and 26); and three second-order factors, which comprise attention (6, 5, 9*, 11, 20, 24, 26 and 28), motor (2, 3, 4, 16, 17, 19, 21, 22, 23, 25 and 30*), non-planning (1*, 7*, 8*, 10*, 12*, 13*, 14, 15*, 18, 27 and 29*). Each item is scored 1, 2, 3 and 4 and summed up within factors, while items with * are reversed and scored 4, 3, 2 and 1. If a total score of 72 or above, is obtained, the participant should be considered as highly impulsive, whereas a total scores ranging between 52 and 71 should be with normal limits for impulsiveness and scores lower than 52 is with in normal limits for impulsiveness and scores lower than 52 are likely to be incredibly over-controlled ^7^.

8. The Multidimensional Health Locus of Control (MHLC) ^9^ consists of 18 items, and is a multidimensional self-report instrument designed to assess an individual’s belief in the locus of control regarding health behaviors. The MHLC includes items relating to internal health locus of control (IHLC) (items1, 6, 8, 12, 13 and 17), powerful others health locus of control (PHLC) (items 3, 5, 7, 10, 14 and 18) and chance health locus of control (CHLC) (items 2, 4, 9, 11, 15 and 16). All items are scored on a 6-point scale from ‘strongly disagree ‘(scored as 1) to ‘strongly agree’ (scored as 6) ^9^.

9. The Tridimensional Personality Questionnaire (TPQ) ^10^ includes 100 items scored true or false. It contains three personality dimensions, namely novelty seeking (NS), harm avoidance (HA) and reward dependence (RD). The novelty seeking subscale inclueds NS1-exploratory excitability versus stoic rigidity (items 2, 4, 9, 11, 40, 43, 85, 93 and 96), NS2-impulsiveness versus reflection (items 30, 46, 48, 50, 56, 81 and 99), NS3-extravagance versus reserve (items 32, 66, 70, 76, 78 and 87), NS4-disorderliness versus regimentation (item13, 16, 21, 22, 24, 28, 35, 60, 62 and 65). Thus, NS total novelty seeking is the sum of 34 items from NS1+NS2+NS3+NS4. The harm avoidance scale includes HA1-anticipatory worry & pessimism versus uninhibited optimism (items 1, 5, 8, 10, 14, 82, 84, 91, 95 and 98), HA2- fear of uncertainty (items 18, 19, 23, 26, 29, 47and 51), HA3-shyness with strangers (items 33, 37, 38, 42, 44, 89 and 100), HA4-fatigability & asthenia (items 49, 54, 57, 59, 63, 68, 69, 73, 75 and 80). Therefore, the total harm avoidance scale includes the sum of HA1+HA2+HA3+HA4. The reward dependence scale consists of: RD1-sentimentality (item 27, 31, 34, 83 and 94), RD2- persistence (item 39, 41, 45, 52, 53, 77, 79, 92 and 97), RD3-attachment (items 3, 6, 7, 12, 15, 64, 67, 74, 86,88 and 90) and RD4-dependence (items 17, 20, 25, 36 and 58). Hence, the total reward dependence includes RD1+RD2+RD3+RD4 ^10^.

10. Schwab and England Activities of Daily Living (ADL)^11^ assesses the capabilities of people with impaired mobility. The scale uses percentages to represent how much effort and dependence on other people need to complete daily chores. It ranges from 0% meaning fully dependant (“Is bedridden and helpless. One is almost completely comatose”), to 100% meaning completely independent (“Able to do all chores without slowness, difficulty or impairment”). The rating may be given by a professional or by the person being tested, and it is easy to use.

References:

1. Georgiev D, Christie R, Torkamani M, Song R, Limousin P, Jahanshahi M. Development and Validation of a Daily Habit Scale. Front Neurosci 2022;16:880023.

2. Hoehn MM, Yahr MD. Parkinsonism: onset, progression and mortality. Neurology 1967;17(5):427-442.

3. Weintraub D, Hoops S, Shea JA, et al. Validation of the questionnaire for impulsive-compulsive disorders in Parkinson's disease. Movement disorders : official journal of the Movement Disorder Society 2009;24(10):1461-1467.

4. Jenkinson C, R. F, V. P, R. G, N. H. The PDQ-8: development and validation of a shortform Parkinson's disease questionnaire. Psychol Health 1997;12:805-814.

5. Zigmond AS, Snaith RP. The hospital anxiety and depression scale. Acta Psychiatr Scand 1983;67(6):361-370.

6. Starkstein SE, Mayberg HS, Preziosi TJ, Andrezejewski P, Leiguarda R, Robinson RG. Reliability, validity, and clinical correlates of apathy in Parkinson's disease. The Journal of neuropsychiatry and clinical neurosciences 1992;4(2):134-139.

7. Knyazev GG, Slobodskaya HR. Personality trait of behavioral inhibition is associated with oscillatory systems reciprocal relationships. International journal of psychophysiology : official journal of the International Organization of Psychophysiology 2003;48(3):247-261.

8. Patton JH, Stanford MS, Barratt ES. Factor structure of the Barratt impulsiveness scale. Journal of clinical psychology 1995;51(6):768-774.

9. Wallston KA, Wallston BS, DeVellis R. Development of the Multidimensional Health Locus of Control (MHLC) Scales. Health Educ Monogr 1978;6(2):160-170.

10. Cloninger CR, Przybeck TR, Svrakic DM. The Tridimensional Personality Questionnaire: U.S. normative data. Psychol Rep 1991;69(3 Pt 1):1047-1057.

11. Schwab RS, England AC. Projection Technique for Evaluating Surgery in Parkinson’s Disease. In: Gillingham FJ, Donaldson IML, editors. Third Symposium on Parkinson’s Disease. Edingurgh: Livingstone; 1969. p. 152-157.
